# Effect of High Temperature and Thermal Cycle of 4043 Al Alloy Manufactured through Continuous Casting Direct Rolling

**DOI:** 10.3390/ma16227176

**Published:** 2023-11-15

**Authors:** Bo-Chin Huang, Fei-Yi Hung

**Affiliations:** Department of Materials Science and Engineering, National Cheng Kung University, Tainan 701, Taiwan; n58081060@ncku.edu.tw

**Keywords:** continuous casting direct rolling (CCDR), 4043 Al alloy, thermal cycle, eutectic Si, microstructure evolution

## Abstract

CCDR 4043 Al alloys are an outstanding candidate for producing mechanical components for automotive or aircraft engines. Two experimental environments—sustained high temperature and repeated heating–cooling—were simulated in the laboratory to replicate the actual operating conditions of engine components. This research investigated the microstructural evolution, mechanical properties, and fracture characteristics of the 4043 Al alloy manufactured through the continuous casting direct rolling (CCDR) process under different post-processing conditions. The CCDR process combines continuous casting, billet heating, and subsequent continuous rolling in a single equipment of production line, enabling the mass production of Al alloy in a cost-effective and energy-efficient manner. In the present work, the 4043 alloy was subjected to two environmental conditions: a sustained high-temperature environment (control group) and a cyclic heating–cooling environment (experimental group). The maximum temperature was set to 200 °C in the experiment. The experimental results show that, in a sustained high temperature working environment, the strength and elongation of the CCDR 4043 Al alloy tend to be stable. The overall effect involves the Al matrix softening and the spheroidization of eutectic Si caused by prolonged exposure to high temperature. This can enhance its ductility while retaining a certain level of mechanical strength. Comparatively, in the working environment of cyclic heating–cooling (thermal cycle), the direction of Si diffusion was different in each cycle, thus leading to the formation of an irregular Ai–Si eutectic structure containing precipitated Si particles of different sizes. The two compositions of Al and Si with very different thermal expansion coefficients may induce defects at the sharp points of Si particles under repeated heating–cooling, thereby reducing the strength and ductility of the material. The results of this work can confirm that the fracture behavior of 4043 Al alloys is obviously controlled by the morphology of the precipitated eutectic Si. In addition, CCDR 4043 Al alloys are not suitable to be used in working environments with a thermal cycle. In practical applications, it is necessary to add traces of special elements or to employ other methods to achieve the purpose of spheroidizing the precipitated eutectic Si and Al–Fe–Si phases to avoid the deterioration of strength and ductility under cyclic heating. To date, no other literature has explored the changes in the microstructure and mechanical properties of CCDR 4043 Al alloys across various time scales under the aforementioned working environments. In summary, the findings provide valuable insights into the effect of thermal conditions on the properties and behavior of CCDR 4043 Al alloys, offering potential applications for it in various engineering fields, such as the automotive and aerospace industries.

## 1. Introduction

In response to growing environmental concerns and the urgent need to combat climate change, automakers have taken aggressive measures to enhance the energy efficiency of their vehicles. This effort aims to reduce carbon emissions and decrease fossil fuel consumption. Utilizing lightweight materials has emerged as a pivotal strategy to address the energy crisis and mitigate environmental pollution [1]. Among these materials, Al–Si alloys stand out as one of the most extensively employed lightweight options. Their popularity is driven by a combination of advantageous characteristics, including exceptional wear resistance, low density, low thermal expansion coefficient at high temperatures, high specific strength, efficient thermal conductivity, excellent castability, and superb corrosion resistance [2,3]. In the automotive sector, Al–Si alloys find application in the manufacturing of crucial engine components, such as cylinder heads, valve tappets, crankshaft covers, pistons, and rocker arms [4]. Furthermore, they serve as a viable alternative to conventional iron-based materials for constructing structural components in automobile bodies, including doors, hoods, and frames. This substitution significantly reduces the overall vehicle weight, making a substantial contribution to energy conservation [5,6]. Beyond their role in the automotive sector, Al–Si alloys also find extensive application in the aerospace industry, particularly in aircraft fuel storage and supply systems, such as fuel tanks and fuel pipelines. These alloys display remarkable corrosion resistance and sealing properties, allowing them to endure high pressures and meet stringent temperature requirements. As a result, they play a crucial role in ensuring the safety and reliability of fuel storage and supply systems in the aviation industry. In summary, the widespread adoption of Al–Si alloys in both the automotive and aerospace sectors highlights their pivotal role in enhancing energy efficiency and reducing environmental impacts. These alloys have become indispensable components in the pursuit of a more sustainable and eco-friendly future. In this study, a 4043 Al alloy with Si content of 5 wt.% was used. The addition of Si can improve the castability and fluidity of the metal and reduce the shrinkage of the metal during the solidification process, so that the 4043 Al alloy has excellent weldability, fluidity, and castability, and at the same time, eutectic Si precipitated at the matrix of Al can effectively improve the wear resistance. The relevant literature has pointed out that Al–Si alloys solidified under traditional cooling conditions are usually composed of dendrites formed by coarse Al grains, eutectic Al–Si structures, and lamellar eutectic Si. This microstructure leads to poor mechanical properties [7,8,9]. Therefore, in recent years, there have been many emerging research attempts to manufacture Al–Si alloys using new process technologies.

Owing to the exceptional fluidity of Al–Si alloys in their molten state and their decreased susceptibility to crack during solidification, research teams have increasingly adopted various advanced production techniques in recent years. These techniques are aimed at fabricating 4043 Al alloys and effectively controlling the grain size, the structure of eutectic Al–Si, and the morphology of the precipitated eutectic Si. These techniques encompass continuous casting direct rolling [10,11,12,13,14], additive manufacturing [15,16,17,18,19], chemical inoculation [7,20,21], and the application of external fields during solidification [22,23]. In this study, we utilized continuous casting direct rolling (CCDR) technology for the fabrication of a 4043 Al alloy. CCDR is a comprehensive process that consolidates various production stages into a single line of equipment. This includes the melting of metal blanks, casting, solidification, rolling, temperature control, coiling, and roll forming [24,25,26,27]. This integrated approach enables the adjustment of production speed and product size to meet specific application requirements while simultaneously enhancing production efficiency and quality [14]. Al–Si alloys, thanks to their low melting point and excellent fluidity, have traditionally found wide usage in welding materials [28,29,30]. Significantly, the outstanding fluidity of Al–Si alloys in their molten state renders them exceptionally well-suited for continuous casting and rolling production when compared to other Al alloys. Therefore, in this study, we employed CCDR technology to manufacture 4043 Al alloy wire rods. Following that, we conducted sustained high-temperature and cyclic heating–cooling experiments to examine the effect of eutectic silicon on the alloy’s mechanical properties and evaluate its suitability for high-temperature working environments.

CCDR enables the mass production of 4043 Al alloy sheets, strips, or coils, which serve as crucial raw materials in the fabrication of fasteners and wear-resistant mechanical components. These materials find extensive application in both the automotive and aerospace industries. Moreover, beyond the advantages of reducing material waste and cutting production costs, CCDR ensures a uniform blank structure while effectively eliminating internal defects. However, 4043 Al alloys are a non-heat-treatable Al alloy, which means that improvement through T6 heat treatment is not feasible. To further enhance the mechanical strength of CCDR 4043 Al alloys, the as-manufactured materials underwent a secondary rolling process at a 60% reduction ratio, successfully avoiding any cracks on the edges of the wire rods. It is important to emphasize that maintaining strength and ductility in high-temperature working environments with repeated cycles of heating and cooling is a crucial research focus, especially given the critical role of these materials as core components in engines, cooling systems, and fuel systems. Previous research has demonstrated that Al–Si alloys typically experience reduced strength and increased elongation after prolonged exposure to high-temperature working environments [4]. Additionally, numerous studies have explored the thermal fatigue life of Al–Si alloys in different test conditions [4,31,32]. In this study, the authors utilized a test model resembling thermal fatigue. Test specimens were repeatedly exposed to both high-temperature and normal room temperature conditions, thereby subjecting them to cyclic heating and cooling, commonly referred to as a thermal cycle [33,34,35,36]. In this comparative test, CCDR 4043 Al alloy specimens were assigned to the control group and exposed to a sustained high-temperature environment, while those placed in a cyclic heating–cooling environment constituted the experimental group. During the experiment, the CCDR 4043 Al alloy specimens were placed in a heating furnace to maintain a temperature of 200 °C and within a thermal cycle apparatus. The subsequent experimental results include an analysis of the microstructure, XRD patterns, and mechanical properties of both groups. Additionally, to gain insights into the effect of eutectic Si morphology and distribution on mechanical properties and damage characteristics, a scanning electronic microprobe (SEM) was employed to investigate the distribution characteristics of precipitated eutectic Si in the transverse section and fractured subsurface of the specimens.

The metal manufacturing industry is now actively pursuing energy efficiency, sustainability, carbon tax compliance, and adherence to ESG (Environmental, Social, and Governance) values [37,38]. CCDR addresses the drawbacks of the high energy consumption and labor intensity inherent in traditional metal manufacturing. Its advantages lie in simplifying the process, reducing labor costs, increasing metal yield, conserving energy, improving the quality of CCDR billets, and facilitating production automation. Utilizing the CCDR process for the production of Al–Si alloys not only promotes environmental friendliness but also reduces carbon emissions while offering competitive raw materials for the automotive manufacturing industry. CCDR technology provides distinctive advantages, complementing the inherent wear resistance, low thermal expansion coefficient, and excellent thermal conductivity of Al–Si alloys, rendering them highly competitive in the market. To ascertain the suitability of CCDR 4043 Al alloys for use as a manufacturing material for engine parts or fasteners, we employed two experimental environments: continuous high temperature and repeated heating–cooling. These simulations aim to replicate the potential scenarios in which mechanical components operate in high-temperature working environments. A subsequent in-depth analysis of mechanical properties testing and microstructure was conducted to determine whether CCDR 4043 Al is qualified for this task, mitigating the risk of unexpected dangers. However, to date, there has been no comprehensive research that systematically investigates the microstructural evolution of Al–Si alloys manufactured through CCDR in a repetitive heating–cooling environment, which leads to changes in mechanical properties. In light of this, this study is essential and presents a practical set of experimental methods to verify the potential application of CCDR Al 4043 alloys in high-temperature working environments. In order to integrate CCDR 4043 Al alloys into the industrial supply chains of the automotive and aerospace sectors as a reliable mechanical component designed for long-term use in various high-temperature working environments, the results presented in this study possess both theoretical significance and practical necessity. They can serve as a crucial benchmark for the industrial application of CCDR Al–Si alloys.

## 2. Materials and Experimental Procedures

**Figure 1:**
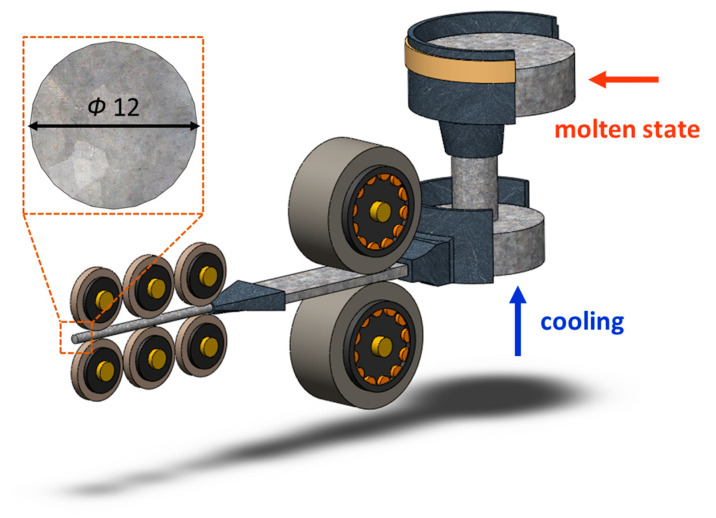
CCDR production equipment.

The CCDR 4043 Al alloy utilized in this study was provided by Ting Sin Co., Ltd. (Tainan, Taiwan). The chemical composition of this alloy is detailed in Table 1, and the production equipment is depicted in Figure 1. 

**Figure 2:**
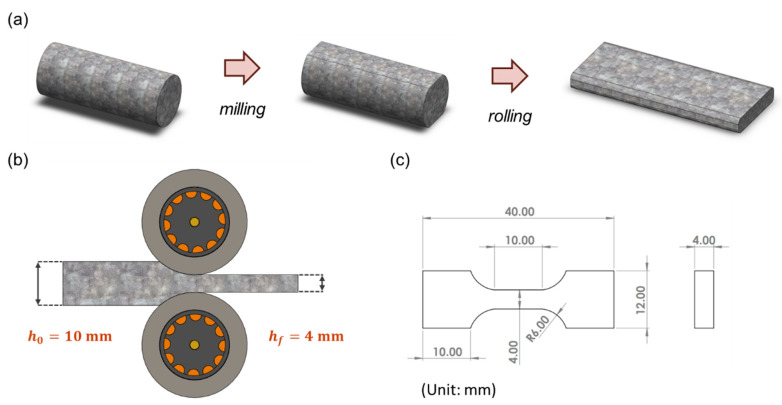
(**a**) Milling and rolling process of specimens. (**b**) Schematic diagram of cold rolling. (**c**) Engineering drawings of tensile test specimens.

The wire rods produced directly from the CCDR production line, in their initial, as-manufactured state, are denoted as ‘specimen F’. As the 4043 Al alloy is a typical non-heat-treatable Al alloy, its mechanical properties cannot be enhanced through T6 heat treatment. Therefore, this study employed strain hardening to improve its mechanical strength. In the pursuit of maximizing mechanical strength through cold working and ensuring the absence of surface rolling cracks to maintain quality, different reduction ratios were considered as experimental parameters during the preparatory experiments. Ultimately, we determined that a 60% reduction ratio yielded the optimal results [10]. The reduction ratio was calculated as the difference between the original thickness and the rolled thickness, divided by the original thickness. The Ø12 wire rods were initially cut and milled into long strips measuring 10 mm in thickness. These strips were then further reduced to 4 mm in thickness through rolling, resulting in specimens referred to as ‘FR’ (the process and equipment are depicted in Figure 2a,b). The strain rate was about 0.03 $s^{-1}$. Subsequently, dog-bone-shaped specimens, as depicted in Figure 2c, were manufactured through wire electrical discharge machining. In the experiment, ASTM E8/E8M [39] was used as the basis for specimen size modification. Stress–strain curves were subsequently recorded using a uniaxial tensile testing machine. (Hung Ta Instrument Co., Ltd., Taichung, Taiwan). Each set of specimens underwent five tests at a tensile rate of 1 mm/min, from which, the average strength and elongation values were calculated. 

**Figure 3:**
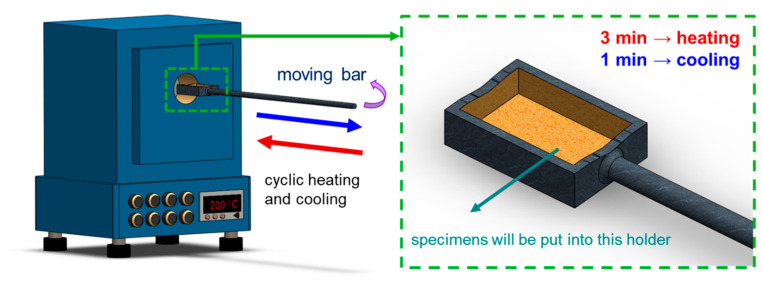
Schematic diagram of self-developed cyclic heating–cooling equipment.

**Figure 4:**
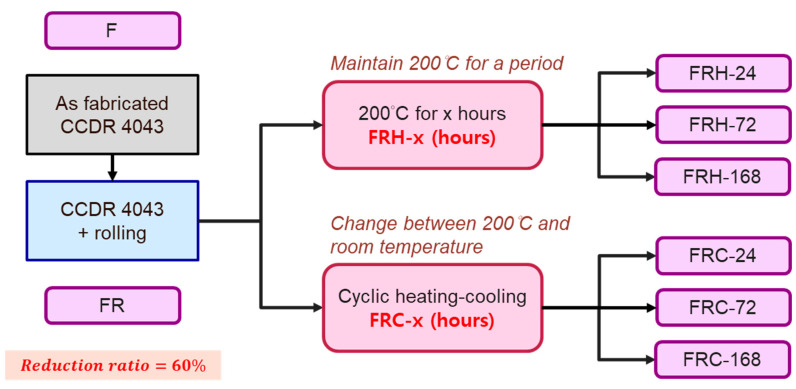
The naming conventions and the workflows of specimen treatments.

The primary objectives of this study centered on two key aspects: (1) examining the effect of sustained high temperatures, and (2) investigating the effects of cyclic heating and cooling (thermal cycle) on the 4043 Al alloy. In the constant high-temperature experiments, the specimens labeled as FR were placed in a heated furnace and maintained at 200 °C for durations of 24, 72, and 168 h, denoted as FRH-24, FRH-72, and FRH-168, respectively. For the thermal cycling experiment, specimen FR was positioned in the thermal cycle equipment as depicted in Figure 3. In this test, the specimen was subjected to a repetitive cycle of heating at 200 °C for 3 min, followed by natural cooling to room temperature (25 °C) for 1 min. This cycle was repeated continuously for periods of 24, 72, and 168 h, designated as FRC-24, FRC-72, and FRC-168, respectively. The naming conventions for the test specimens are summarized in Table 2, and the process framework is illustrated in Figure 4. The setting of 200 °C as the maximum temperature limit is based on its slight elevation above the artificial aging temperature of typical Al alloys. Beyond this threshold, unpredictable changes in the microstructure may occur, potentially affecting the mechanical properties. Moreover, under elevated temperature conditions, the prolonged use of Al–Si alloys becomes less favorable. In such circumstances, opting for metals with higher temperature resistance, even if sacrificing some lightweight advantages, would be a prudent decision.

To examine the metallurgical structure, the specimens mentioned above underwent a sequential process. They were initially ground using SiC sandpaper (3M Company, Maplewood, NJ, USA) with grit sizes ranging from 120 to 4000#. Affixing sandpaper to the rotary disk of the grinding and polishing equipment, the metallographic specimens were then gently pressed on the sandpaper with fingers. Subsequently, the specimens were polished using aqueous solutions of Al_2_O_3_ and SiO_2_. Finally, they were immersed in Keller’s corrosive solution, composed of 2 mL HF, 3 mL HCl, 5 mL HNO_3_, and 190 mL H_2_O, for approximately 20–25 s. The specimens were then observed under an optical microscope (Olympus BX41M-LED, Tokyo, Japan). This process allowed for the assessment of microstructure characteristics across different sections. In this article, the plane that is perpendicular to the secondary rolling is referred to as the ‘transverse section’, while the plane parallel to the secondary rolling is termed the ‘axial section’. Material hardness was analyzed using a Rockwell hardness testing machine (Mitutoyo AR-10 Hardness Testing Machine, Sakado, Japan), following the HRF test specification (in compliance with ASTM E18-20 [40]). A steel ball indenter with a diameter of 1/16 inches was used as the load source, applying a force of 60 kgf. The hardness measurements were recorded for both the transverse and axial sections of the test specimen. The constituent phases were identified using an X-ray diffractometer (Bruker, Billerica, MA, USA) operating under the following conditions: a voltage of 45 kV, a current of 100 MA, a 2θ range from 20 to 90 degrees, and a scanning speed of 2.4 degrees per minute. The fracture surfaces and precipitated eutectic Si were analyzed using a scanning electron microscope (SEM) (Hitachi SU-5000, Hitachi, Tokyo, Japan) equipped with an energy-dispersive spectroscopy (EDS) instrument. The accelerating voltage of the SEM was from 0.5 KV to 30 KV, and the working distance of SEM was about 160 mm, which was adjusted according to the height of the specimens. To investigate the effect of the distribution of Si and precipitated crystalline Si morphology on the mechanical properties of the 4043 Al alloy, specimens were extracted from the specimens listed in Table 2. These specimens, taken from the transverse section, were subjected to precision grinding and polishing. The elemental distribution within the transverse cross-section was analyzed using a high-resolution electron probe microanalyzer (EPMA) (JEOL JXA-8530 F, Tokyo, Japan). Additionally, specimens FR, FRH-168, and FRC-168 were specifically chosen for an analysis of the fracture subsurface. Finally, to thoroughly evaluate the atomic-scale precipitation behavior within the internal structure of the 4043 Al alloy under two distinct operating environments (sustained high temperatures and thermal cycle), samples were extracted from specimens FRH-168 and FRC-168 using a focused ion beam (FIB) (Hitachi NX2000, Tokyo, Japan). Phase identification was conducted using a transmission electron microscope (TEM) (FEI Tecnai F20 G2 Field-emission Scanning Transmission Electron Microscope, Hillsboro, OR, USA).

## 3. Results and Discussion

### 3.1. Microstructure Evolution

**Figure 5:**
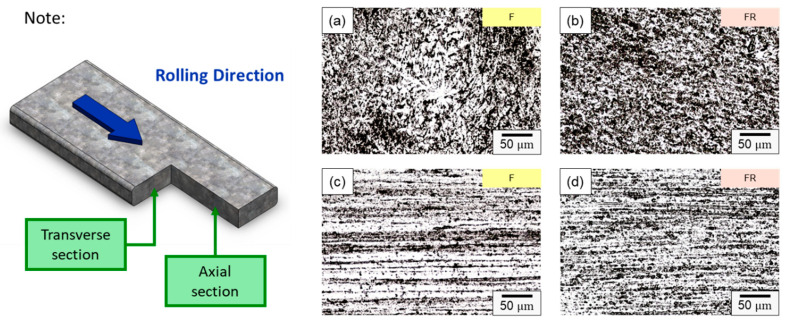
Microstructure of specimens F in (**a**) transverse and (**c**) axial sections, and specimens FR in (**b**) transverse and (**d**) axial sections.

The microstructure of the CCDR 4043 Al alloy in its as-manufactured state and after secondary rolling is depicted in Figure 5. The CCDR process induced a texture effect, resulting in distinct microstructures in the directions perpendicular and parallel to the rolling process. In Figure 5a,b, the microstructures of specimens F and FR in the transverse section are presented.

**Figure 6:**
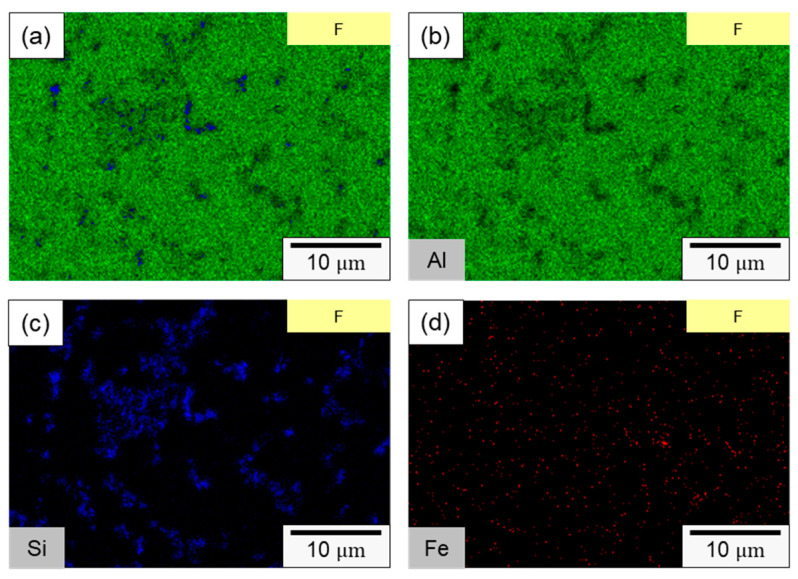
SEM element mapping of specimen F: (**a**) overlaying image, (**b**) Al, (**c**) Si, (**d**) Fe.

In specimen F, the primary components consisted of α–Al dendrites and a dark eutectic Al–Si structure. Following the second rolling process (specimen FR), the eutectic Al–Si structure underwent noticeable deformation, forming a Si-rich conglomerate aggregation. Figure 5c,d present the microstructure of the axial section for specimens F and FR. In both instances, Si-rich aggregates that formed along the rolling direction are evident, alongside point-like eutectic Al–Si phases distributed within the matrix. It is worth noting that after the secondary rolling process, specimen FR exhibited a more pronounced long island structure compared to specimen F. According to the literature, eutectic Si in Al–Si alloys precipitates as pure Si crystals [41,42], taking the form of flakes or granules. It also combines to form a eutectic Al–Si structure with Al [43]. This eutectic Al–Si structure is distributed within the darker part of the matrix, and is composed of needles, rods, and branches. In Figure 6, a more detailed analysis of the elemental distribution in the transverse section, performed using EDS, confirmed that the dark regions in Figure 5a,b primarily consisted of highly aggregated Si, with a small amount of Si solidly dissolved in the matrix. Figure 6d demonstrates that Fe was relatively uniformly distributed, with only a few aggregates. The presence of iron in the Al–Si alloy system resulted in the formation of several intermetallic phases. The existing literature suggests that a significant portion of Fe is solidly dissolved within the α–Al, while some of it forms the Al–Fe–Si phase in conjunction with Al and Si [44,45]. The sharp corners and incoherent interface of the Al–Fe–Si act as stress concentrators in the α–Al matrix, leading to severe brittleness in the final Al–Si alloy casting. As the presence of Fe is unavoidable within the 4043 Al alloy, subsequent experiments will meticulously examine the changes in the distribution of Fe using EPMA.

**Figure 7:**
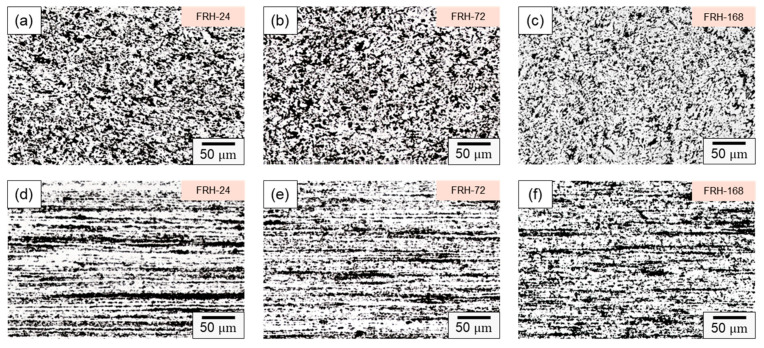
Microstructure of specimen FRH-24 in (**a**) transverse and (**d**) axial sections; specimen FRH-72 in (**b**) transverse and (**e**) axial sections; and specimen FRH-168 in (**c**) transverse and (**f**) axial sections.

Figure 7 illustrates the microstructure evolution of the CCDR 4043 Al alloy at various time intervals of sustained high-temperature exposure. Figure 7a–c reveal that in the transverse section, Si-rich agglomerates gradually diffused and underwent morphological changes at elevated temperatures. Simultaneously, the eutectic Al–Si structure gradually transformed into needle-like and dendritic shapes, closely resembling the original specimen F. Figure 7d–f shows that in the axial section, the microstructure did not change significantly after a long period of high temperature treatment. 

**Figure 8:**
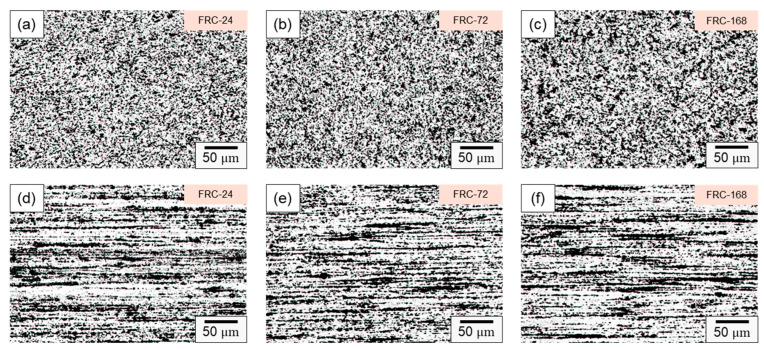
Microstructure of specimen FRC-24 in (**a**) transverse and (**d**) axial sections; specimen FRC-72 in (**b**) transverse and (**e**) axial sections; and specimen FRC-168 in (**c**) transverse and (**f**) axial sections.

Figure 8 presents the microstructure evolution of the CCDR 4043 Al alloy within a cyclic heating–cooling environment at various time intervals. In Figure 8a–c, it is evident that, within the transverse section, the Si-rich agglomerates, initially formed by the secondary rolling, evolved into various structures, including needle-shaped, dendritic, point-like, and irregular forms after varying numbers of thermal cycles. This phenomenon arises from the fact that, under repeated heating–cooling conditions, the Si element spreads out and combines with Al, forming various Al–Si structures. These Al–Si precipitates, which appear as dark-colored regions in the optical microscope image, primarily consist of lamellar Si and Si particles. The sharp edges and corners of the Si phase are particularly susceptible to generating uneven stress, thus becoming the starting point for material failure under stress. Consequently, the control of Si particle morphology and the Al–Si phase to enhance mechanical properties is a crucial metallurgical challenge in the context of Al–Si alloys [46,47]. Figure 8d–f depict the microstructure within the axial section, revealing that the influence of the cyclic heating–cooling environment on the microstructure was relatively subtle. The Si-rich aggregates maintained their original long island shape, with only a portion of the point aggregates re-dissolved into the long island structure due to Si diffusion or formed coarser clusters of Si particle aggregation.

**Figure 9:**
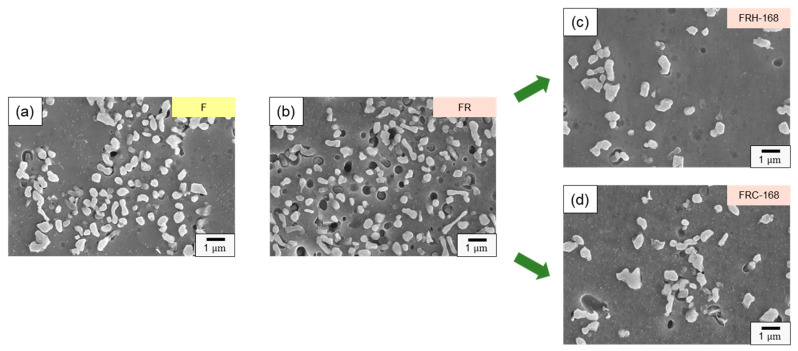
Evolution of precipitated Si particles of specimens (**a**) F, (**b**) FR, (**c**) FRH-168, and (**d**) FRC-168.

Figure 9 presents the distribution and size of internal Si particles in the eutectic Al–Si phases within specimens F, FR, FRH-168, and FRC-168. The size distribution of Si particles was about 0.2–1.5 μm. Due to significant variations in the Si particle content depending on the sampling location, we focused solely on discussing the changes in morphology and size in Figure 9. Figure 9a,b illustrate the influence of cold workability on Si precipitation in the pristine state of the 4043 Al alloy specimens after the secondary rolling. The experimental results confirm that both α–Al dendrites and the eutectic Al–Si structures underwent changes due to compressive stress. Furthermore, the internal flaky and granular Si within the eutectic Al–Si phase transformed into finer Si particles under pressure. Figure 9c,d showcase the microstructure of the CCDR 4043 Al alloy after 168 h of sustained high-temperature and repeated heating–cooling conditions following the secondary rolling. As shown in Figure 9c, prolonged exposure to 200 °C promoted Si diffusion, allowing the Si within the matrix to merge with the originally precipitated Si particles found in specimen FR, resulting in a significant increase in particle size. Conversely, under thermal cycle conditions, repeated heating–cooling led to varying directions of Si diffusion within the microstructure, resulting in a greater difference in sizes between Si particles.

**Figure 10:**
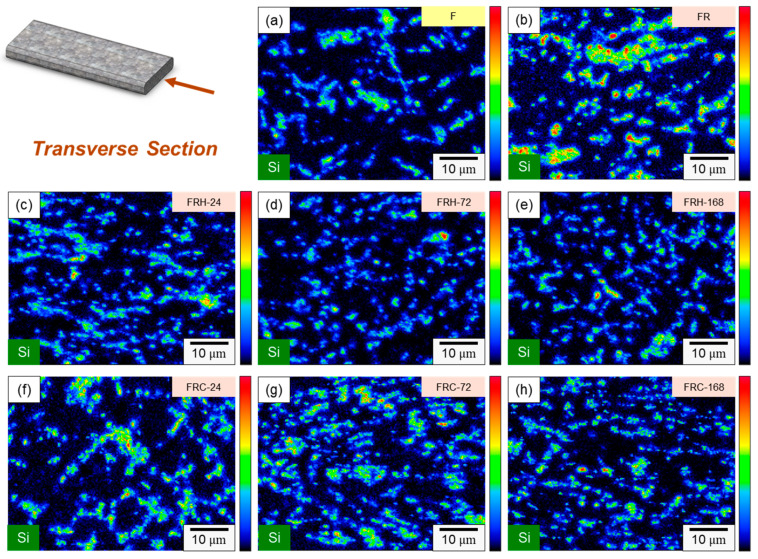
Si distribution evolution of specimens (**a**) F, (**b**) FR, (**c**) FRH-24, (**d**) FRH-72, (**e**) FRH-168, (**f**) FRC-24, (**g**) FRC-72 and (**h**) FRC-168.

Figure 10 provides magnified 2000× transverse sections of the eight sets of specimens listed in Table 2, allowing for a detailed investigation of the Si distribution evolution under various post-processing conditions. The first, second, and third rows of Figure 10 depict the effect of cold working, sustained high temperature, and thermal cycling on the Si distribution in the microstructure, respectively. Figure 10a,b reveal the formation of clustered aggregates of needle-shaped eutectic Si and Si distributed within the eutectic Al–Si phase due to compression. In Figure 10c–e, it is evident that during sustained high-temperature conditions, a portion of the Si in the eutectic Al–Si phase re-solidified within the α–Al matrix, while another portion gradually transformed into precipitated Si in the form of rods and particles. Figure 10f–h confirm that the Si within the Si-rich clusters formed unevenly sized particles and irregular structures during thermal cycling. Comparing Figure 9c,d with Figure 10e,h, the sustained high temperature exposure facilitated the formation of spheroidized eutectic Si and short rod-shaped Si clusters, creating a uniform network that enhanced material ductility. In contrast, the cyclic heating–cooling conditions tended to generate irregular Si particles. Therefore, it can be deduced that the thermal expansion coefficients of Al and Si in 4043 Al alloys vary significantly under repeated heating–cooling conditions, inducing defects at the sharp corners of Si particles, leading to reduced strength and decreased material ductility.

**Figure 11:**
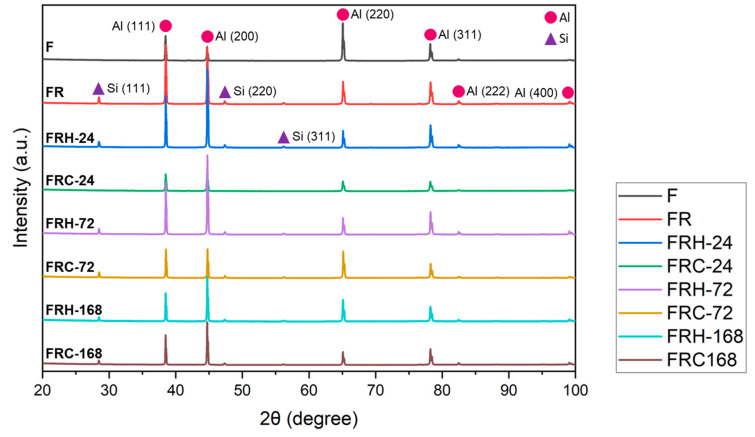
X-ray diffraction pattern of all specimens.

Figure 11 displays the X-ray diffraction (XRD) pattern of the CCDR 4043 Al alloy under the various post-treatment conditions. The diffraction pattern primarily consisted of Al and Si crystal planes with different orientations. Notably, the change in crystal orientation due to secondary rolling resulted in a significant increase in peak intensity for Al(111), Al(200), Al(222), and Al(400), accompanied by a decrease in Al(220) peak intensity. Simultaneously, the eutectic Al–Si phase formed Si-rich agglomerates, leading to the appearance of Si(111), Si(220), and Si(311) peaks in the diffraction pattern. In the sustained high-temperature environment, the Si diffusion and homogenization effects within the 4043 Al alloy resulted in a slight decreasing trend in both Al and Si peak intensities. This decreasing trend became more pronounced with longer temperature holding times. In summary, in a cyclic heating–cooling environment, the Si diffusion direction and path vary in each cycle, leading to less significant drops in both Al and Si peaks.

### 3.2. Hardness and Mechanical Properties

**Figure 12:**
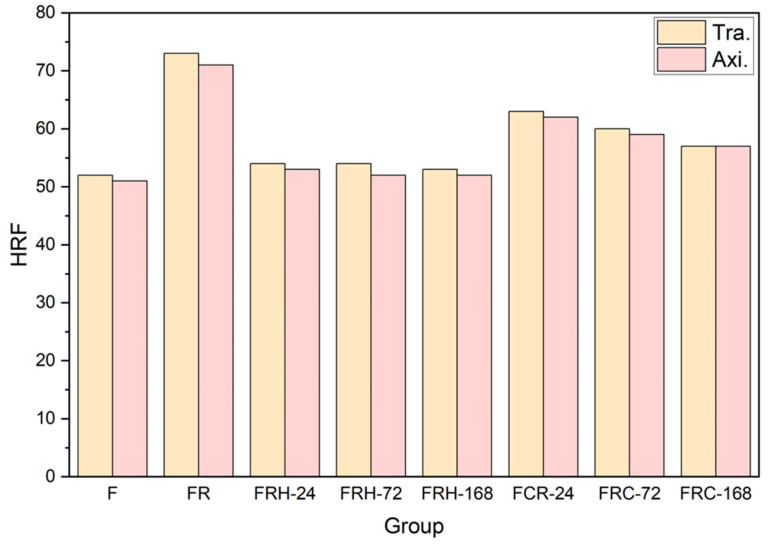
Rockwell hardness (HRF) of all specimens.

Figure 12 illustrates the hardness of the CCDR 4043 Al alloy under the various post-processing conditions. The original 4043 Al alloy (specimen F) exhibited a hardness of HRF 52 in the transverse section and HRF 51 in the axial section. A subsequent secondary rolling led to work hardening and an accumulation of hard silicon, resulting in increased hardness. Specifically, the transverse section and axial section hardness for specimen FR measured HRF 73 and HRF 71, respectively. The transverse section hardness decreased from HRF 73 to HRF 53 and the axial hardness decreased from HRF 71 to HRF 52 after 168 h of sustained high temperature exposure. After subjecting the material to 168 h of the sustained high temperature treatment at 200 °C, we observed a decrease in hardness. This constant high temperature effectively eliminated the dislocations caused by cold rolling and softened the matrix. The hardness of the specimens stabilizes after 24 h, with minimal changes over the following 168 h. Furthermore, when subjected to cyclic heating–cooling conditions, both the transverse and axial hardness exhibited a gradual decline. The thermal cycle did not provide a stable driving force for grain growth, leading to the multidirectional diffusion of silicon evolving from agglomerates to needle rods. Within the 168 h timeframe, the hardness of the test piece did not reach a stable value, consistent with the observations in Figure 10f–h, indicating that silicon primarily existed in irregular shapes within the eutectic phase.

**Figure 13:**
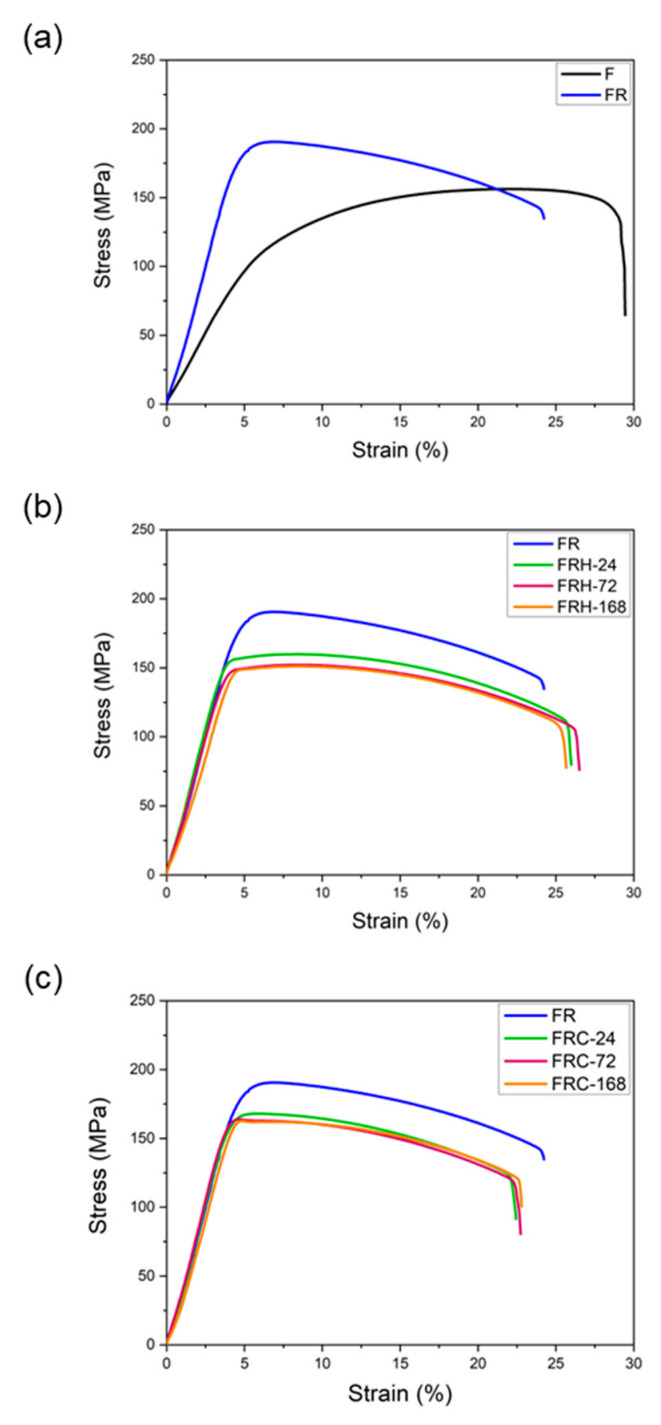
Stress–strain curve comparison of (**a**) specimens F and FR; (**b**) specimens FR, FRH-24, FRH-72, and FRH-168; (**c**) specimens FR, FRC-24, FRC-72, and FRC-168.

**Figure 14:**
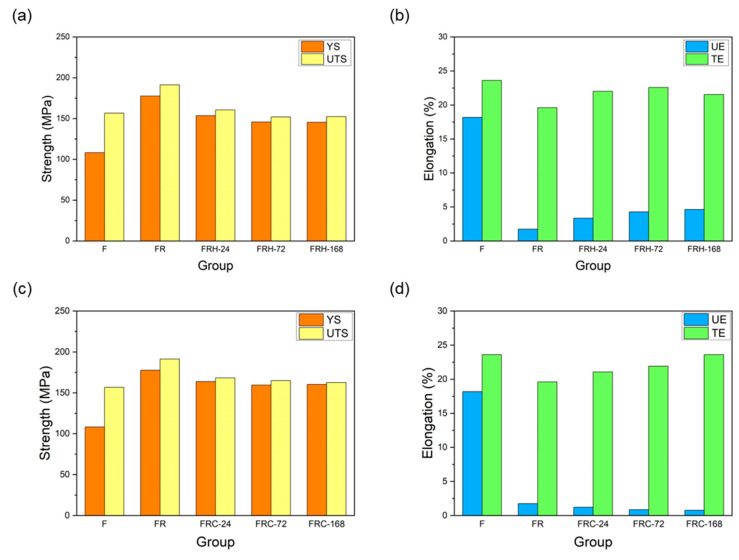
Effect of sustained high temperature on (**a**) strength and (**b**) elongation. Effect of thermal cycling on (**c**) strength and (**d**) elongation.

Figure 13a confirms that secondary rolling with a 60% reduction rate can enhance both the yield strength and ultimate tensile strength, while maintaining control over uniform and total elongation within acceptable limits. Figure 13b,c, respectively, illustrate the changes in the stress–strain curves of specimen FR under two different working conditions: sustained high temperature and cyclic heating–cooling. The individual effects of each working condition on the strength and elongation of the CCDR 4043 Al alloy after secondary rolling are summarized in Figure 14. It is observed that both sustained high temperature and thermal cycling conditions resulted in a reduction in strength and ultimate tensile strength. The most noteworthy distinction between Figure 13b,c is the matrix softening and the globularization of Si particles induced by sustained high temperature treatment, which contributes to the improved ductility of the 4043 Al alloy [46,48]. Figure 14b reveals that the elongation of specimens FRH-24, FRH-72, and FRH-168 surpassed that of specimen FR. In contrast, cyclic heating–cooling failed to provide sufficient time and stable diffusion for the homogenization of the internal grains in the CCDR 4043 Al alloy. Moreover, it is worth noting that the coefficient of thermal expansion for pure Al is approximately 6.6 times that of Si [49,50]. The cyclic stresses induced by repeated thermal expansion and cold contraction of hard silicon within a soft Al matrix can create defects at the interface between Al and silicon, which can, in turn, impact both strength and elongation.

### 3.3. Fracture Behavior

**Figure 15:**
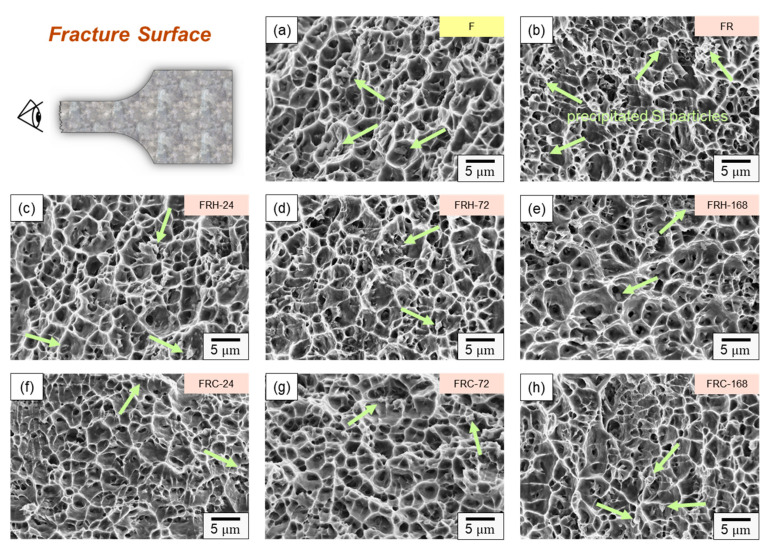
Fracture morphology of specimens (**a**) F, (**b**) FR, (**c**) FRH-24, (**d**) FRH-72, (**e**) FRH-168, (**f**) FRC-24, (**g**) FRC-72, and (**h**) FRC-168 (Note: The precipitated Si particles on the fractured surface are indicated by bright green arrows).

Figure 15 illustrates the fracture surface characteristics of the specimens listed in Table 2 after undergoing room temperature tensile testing. All specimens exhibited typical ductile fractures dominated by dimple characteristics. The size of these dimples on the fracture surface closely correlated with the material’s fracture behavior. A comparison of Figure 15a,b reveals that specimen FR had smaller dimples than specimen F. Larger dimples typically indicate enhanced material ductility since they signify substantial plastic deformation before fracture, resulting in increased elongation. This observation aligns with the findings presented in Figure 13a. Moving on to Figure 15c–e, it is evident that an increase in the holding temperature at 200 °C led to larger dimples on the fracture surface and, concurrently, a decrease in the number of Si particles. These results suggest that spheroidized Si plays a role in restraining crack propagation within the material. Figure 15f–h displays dimples of varying sizes and a higher density of the ridge structure surrounding the dimples. This indicates that the material experienced crack initiation from multiple points under stress, and a greater number of Si particles was evident on the fracture surfaces. When comparing Figure 15e,h, the latter depicts dimples of different sizes, along with numerous tearing ridges and Si particles. This pattern implies that cracks propagated along the Al–Si interface, a crucial factor contributing to the diminished strength and elongation of the 4043 Al alloy under thermal cycling experimental conditions.

**Figure 16:**
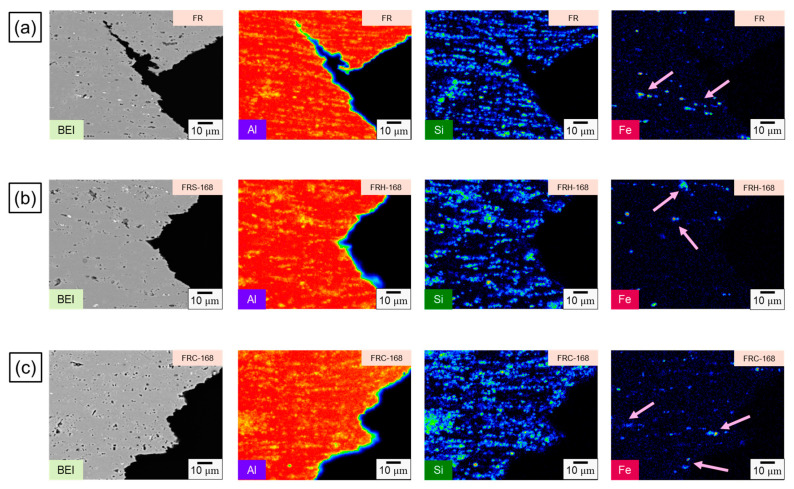
EPMA scanning element mapping of specimens (**a**) FR, (**b**) FRH-168, and (**c**) FRC-168. The first, second and third rows of the Figure 16 represent the BEI and the distribution of Al, Si and Fe of specimens FR, FRH-168 and FRC-168 respectively. Fe-rich accumulation on the fracture subsurface is shown by the pink arrows.

To elucidate the fracture mechanism of the CCDR 4043 Al alloy, we further conducted a comparison of the elemental distributions on the fracture subsurface of specimens FR, FRH-168, and FRC-168, as depicted in Figure 16. Fe influences crack growth by precipitating a needle-like Al–Fe–Si phase within the Al–Si alloy [51,52]. Previous studies have indicated that the fracture of Al–Si casting alloys with trace amounts of Mg and Cu initiates in the interdendritic regions that contain brittle, Fe-rich intermetallic compounds [53,54]. As such, the phases that affect the fracture behavior in our present study encompassed (1) eutectic Si and (2) Al–Fe–Si phases. The combined effect of the morphology and base softening of these two phases is a pivotal factor in determining the material’s strength and elongation [48]. Comparing the evolution of Si distribution shown in Figure 16a–c, we observed that the thick, island-like precipitated Si, originating from the secondary rolling, gradually transformed into spheroidal and elliptical aggregates after 168 h of exposure to a 200 °C holding temperature. This transformation aided in further impedance of crack propagation. Conversely, after 168 h of cyclic heating and cooling, Si was unevenly distributed in long strips and clusters, resulting in limited ductility improvement and even inducing crack growth at the Al–Si interface, leading to a diminished strength under load. Notably, we can infer from Figure 16a,c that Si closely corresponds to the fracture subsurface profile in BEI, whereas in Figure 16b, Si exhibited a discontinuous distribution at the fracture edge.

**Figure 17:**
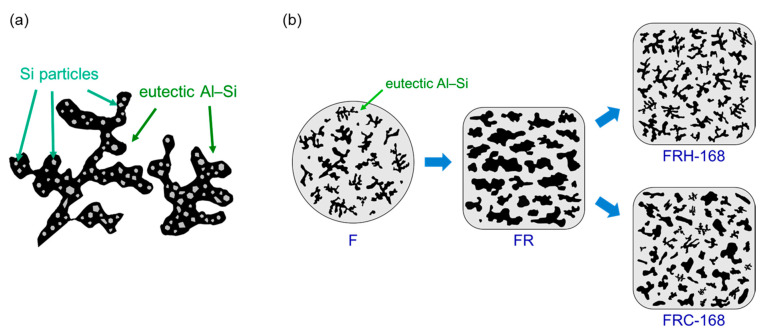
Schematic diagrams illustrating (**a**) the Al–Si eutectic structures and (**b**) the morphological evolution of precipitated Si.

The preceding discussion substantiates that the fracture behavior of the 4043 Al alloy is fundamentally governed by the morphology of precipitated Si. On the other hand, the evolution of the Fe distribution in Figure 16a–c sheds light on the needle-like Al–Fe–Si phase within specimen FR. This phase underwent morphological transformations when exposed to a 168 h holding period at 200 °C, effectively mitigating stress concentration points typically induced by the sharp edges of hard secondary phases [55]. Simultaneously, the spheroidized Fe-rich phase acts as an impediment to crack growth [56,57]. However, following 168 h of cyclic heating and cooling, the original needle-like precipitated Al–Fe–Si phase morphed into short rod-like and point-like aggregations with an uneven distribution. This led to non-uniform stress distribution within the material when subjected to mechanical loads. Figure 17a illustrates the dendritic Al–Si eutectic structure with embedded precipitated Si particles. Figure 17b illustrates the evolution of the eutectic Al–Si structure’s morphology in the transverse section of specimens F, FR, FRH-168, and FRC-168.

### 3.4. TEM Diffraction Analysis

**Figure 18:**
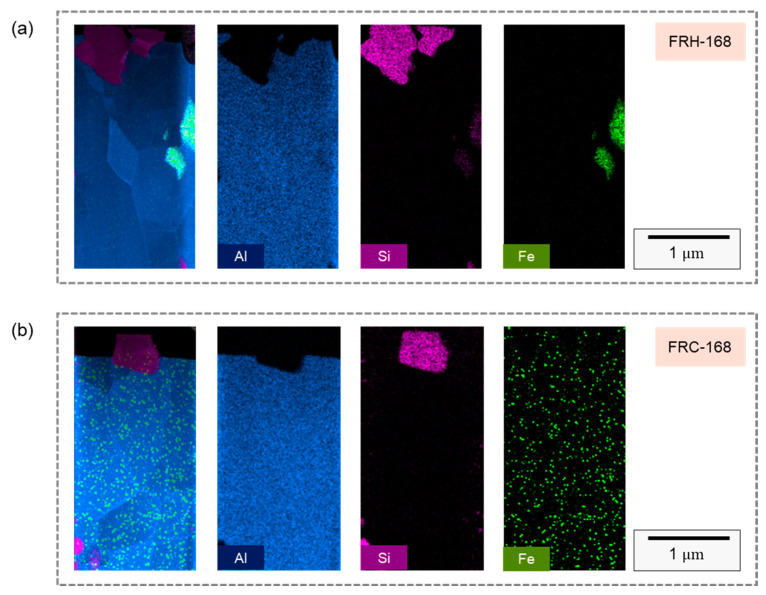
TEM analysis of EDS mapping in FIB slice section of specimens (**a**) FRH-168 and (**b**) FRC-168.

Figure 18a,b illustrate the elemental distribution along the FIB sampling profiles for specimen FRH-168 and specimen FRC-168, respectively, revealing the presence of micrometer-scale Si particles in both cases. Moreover, due to the sampling location, the precipitated Al–Fe–Si phase formed by Fe aggregation can be discerned within specimen FRH-168. In contrast, specimen FRC-168 was not sampled in the Fe-rich region, making the Fe signal source in Figure 18b comprise Fe originating from a solid solution within the Al matrix and background signals. Subsequently, Si particle diffraction patterns were employed to examine the crystallization behavior, as showcased in Figure 18.

**Figure 19:**
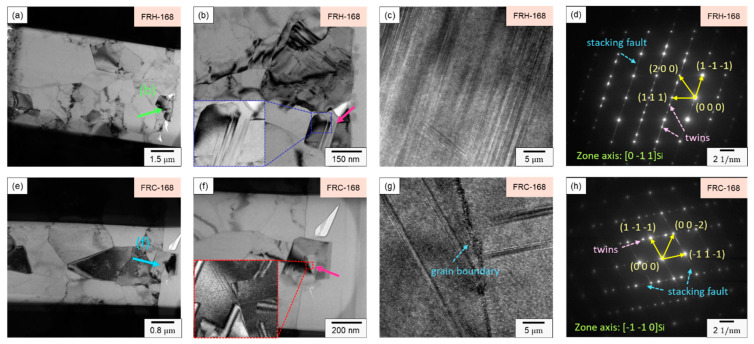
(**a**,**e**) Bright field TEM images; (**b**,**f**) enlarged view of Si particles; (**c**,**g**) HRTEM images and (**d**,**h**) SAED patterns of specimens FRH-168 and FRC-168.

In Figure 18c, the HRTEM image of specimen FRH-168 reveals a high-density stacking of atoms, indicating that elevated temperatures and extended durations foster stacking faults within the 4043 Al alloy Si particles. Figure 19d represents the diffraction pattern corresponding to Figure 19c, revealing the presence of stacking faults and twins along multiple directions. In Figure 19g, the HRTEM image of specimen FRC-168 displays clearly visible curved grain boundaries. The distinct Si diffusion directions during heating and cooling, coupled with the inability of thermal cycles to provide a stable driving force for grain growth, led Si to solidly dissolve in the Al matrix and precipitate from various nucleation points. Consequently, these Si particles coalesced to form Si particles, with some stacking faults evident within the eutectic Si on either side of the grain boundary. Figure 18h corresponds to the diffraction pattern in Figure 19g. Comparing Figure 19c,d and Figure 19g,h, the experimental results indicate that prolonged exposure to high temperatures results in the development of more stacking faults and defects within the eutectic Si.

**Figure 20:**
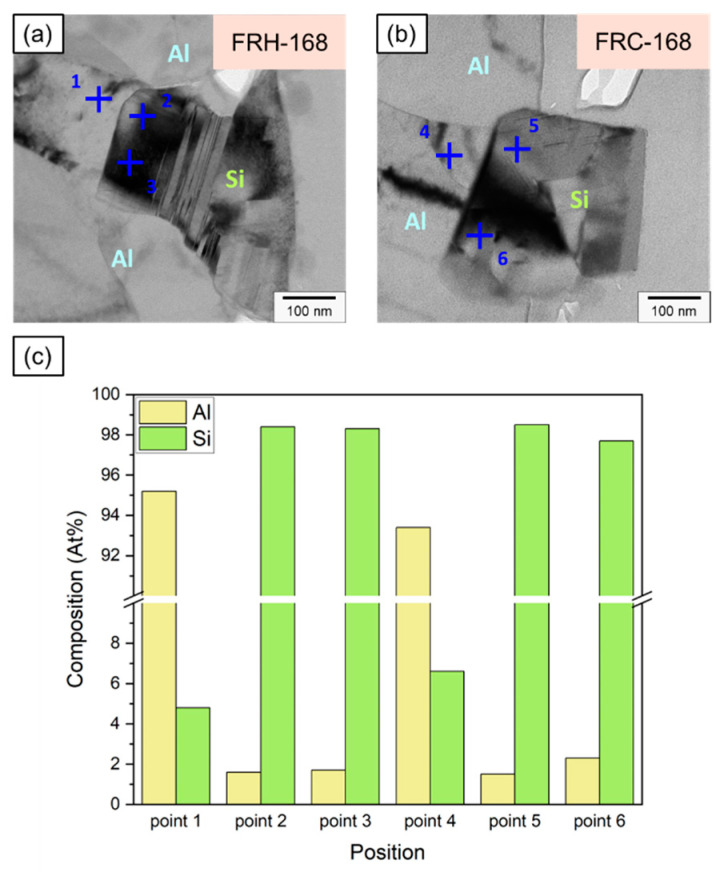
Position marking for EDS analysis near Al–Si interface of specimens (**a**) FRH-168 and (**b**) FRC-168. (**c**) Composition analysis of Al and Si at each point.

Figure 20a,b illustrate the Al and Si compositions on both sides of the Al–Si interface, detected using EDX, for specimens FRH-168 and FRC-168. Figure 20c confirms that the Si concentration in the Al matrix of specimen FRH-168 was approximately 2 a.t% lower than that of FRC-168 (compare points 1 and 4). This observation aligns with the results in Figure 10. The prolonged exposure to high temperatures induced the diffusion of solid solution Si from the matrix into the precipitated Si, resulting in a slightly reduced Si content in the α–Al. Conversely, the Al and Si contents in the Si particles show only minor differences between the two specimens.

**Figure 21:**
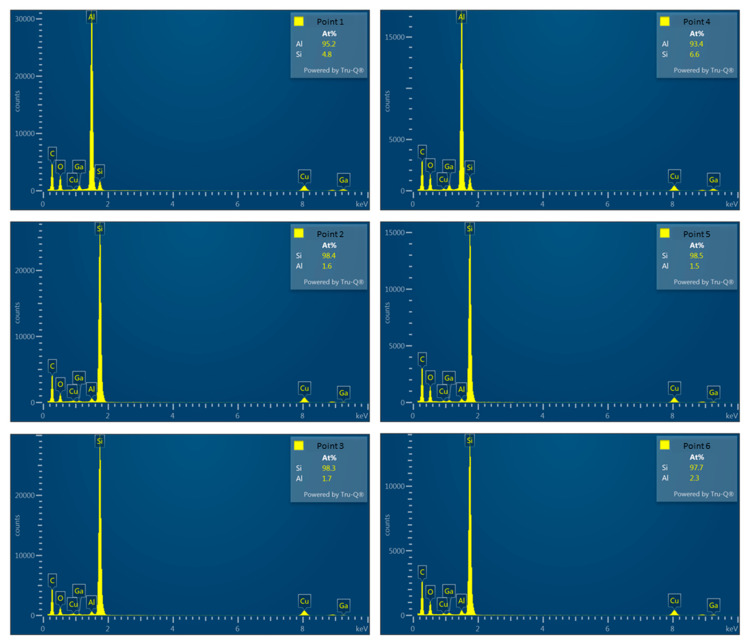
Corresponding EDS analysis of each point.

It is worth noting that past studies on Al–Si alloys have shown that Si is primarily distributed in Al bases as eutectic Si [8,9], which takes the form of flakes and consists of pure Si [58,59]. Figure 21 and Table 3 present the EDX detection results for points 1–6, revealing the presence of approximately 1.6–2.3 at.% of Al solid solution within Si crystals at points 2, 3, 5, and 6. This result may be attributed to scattered electrons impacting the convex areas left by FIB when grinding the thin area on both sides of the TEM specimen, thereby generating weak X-ray signals characteristic of Al. Viewing this result from another perspective, Jiqiang Chen et al. claimed the presence of trace amounts of Al precipitates in the eutectic Si phase in previous research [60]. Nevertheless, the current study was unable to validate this view, and we believe that the precipitated Si particles in the 4043 Al alloy consist of pure Si.

### 3.5. Application Potential and Future Work

The 4043 Al alloy belongs to the 4xxx series of Al alloys and is known for its exceptional characteristics. It exhibits high fluidity at elevated temperatures, resists solidification cracks, and maintains crack resistance during cold processing. As a result, it is well-suited for mass production using the CCDR process compared to other Al alloy types. In this study, we utilized CCDR to produce 4043 Al alloy wire rod coils with the primary goal of developing lightweight, high-quality, and cost-effective raw materials. These materials find applications in the production of fasteners and wear-resistant mechanical components, such as screws, nuts, bolts, sockets, and pins. It is worth noting that the 4043 Al alloy wire rod coils used in our research have achieved IATF16949 certification. This global quality management system certification is crucial for the automotive industry, further underscoring the alloy’s potential applicability in this sector.

Furthermore, our experimental findings demonstrated that, in a thermal cycling environment, the inability to achieve spheroidized eutectic Si precipitation and uniform Si particle distribution within the 4043 Al alloy led to a gradual reduction in strength and ductility during repeated heating–cooling cycles. This finding aligns with the observation in Figure 16b, where Si was discontinuously distributed at the fracture edge, indicating that the crack bypasses the spheroidized Si particles during propagation. A closer examination of the third picture in Figure 16a–c can further validate our inference regarding the Si distribution at the edge. As depicted in the evolution process in Figure 17b, the size of the Al–Si eutectic structure in FRC differed significantly from that in FRH. The repeated heating and cooling cycles hindered the effective smoothening and spheroidizing of sharp corners in the Si particles within the structure. In our forthcoming research, we aim to enhance the application potential of CCDR 4043 Al alloys by introducing trace amounts of Sr and Mg to control Si nucleation and precipitation strengthening [61].

## 4. Conclusions

In this study, a 4043 Al alloy was fabricated by the continuous casting and rolling technique and cold rolling was applied to enhance the mechanical strength. The subsequent testing included (1) sustained high temperature and (2) thermal cycle maintenance for 24, 72, and 168 h to analyze the changes in microstructure and mechanical properties. Finally, a comparison of the Si crystallization behavior of specimens FRH-168 and FRC-168 was made and the following conclusions were obtained:

(1) Prolonged exposure to 200 °C leads to Si diffusion within the matrix of the 4043 Al alloy, resulting in Si particle coarsening. Additionally, sustained high temperatures eliminate dislocations induced by cold rolling, rendering the matrix more malleable. The uniform network of spheroidized Si and small rod-shaped Si clusters enhances the material’s ductility and reduces the concentration effect of hard-phase stress.
(2) The cyclic heating–cooling environment induces the formation of Al–Si eutectic structures of varying sizes within the 4043 Al alloy, attributed to the diverse directions of the diffusion driving force in each cycle. Sustained high temperature treatment, in comparison with repeated heating and cooling, promotes the spheroidization of more Si particles, particularly at sharp corners.
(3) The thermal expansion coefficient of Al is approximately 6.6 times that of Si. Repeated cycles of thermal expansion and contraction can readily induce cyclic stress, leading to defects at the Al–Si interface. This, in turn, causes cracks to propagate along the Al–Si interface when the 4043 Al alloy is under load, resulting in a deterioration in strength and elongation.
(4) When comparing the crystallization behavior of Si under long-term temperature holding and thermal cycling conditions, it becomes evident that a sustained high-temperature environment results in high-density stacking faults and twin crystals forming within the Si particles. In contrast, thermal cycling does not offer a stable driving force for Si growth. Instead, solid solution Si, dissolved in the Al matrix, tends to precipitate from multiple nucleation points, leading to the observation of grain boundaries and dislocations within the Si structure.
(5) In comparison to repeated heating and cooling conditions, mechanical components manufactured using a CCDR 4043 Al alloy exhibit greater stability under sustained high-temperature conditions. Exploring alternative methods to achieve the spheroidization of precipitated Si and the uniform distribution of Al–Si eutectic structures within the microstructure of Al–Si alloy in a thermal cycle working environment is a crucial engineering challenge that requires further investigation in future research.

## Figures and Tables

**Table 1:** The chemical composition of the 4043 Al alloy used in this study.

	Al	Si	Fe	Cu	Mn	Mg	Zn	Ti
Composition (wt.%)	Bal.	4.5–6.0	≤0.8	≤0.30	≤0.05	≤0.05	≤0.10	≤0.20
This study	Bal.	4.86	0.23	0.079	0.003	0.002	0.017	0.02

**Table 2:** The naming principles and post-processing of specimens.

Name	Post-Processing
F	As-manufactured through CCDR
FR	Specimen F rolled at a reduction ratio of 60%
FRH-24	Specimen FR in an environment of 200 °C for 24 h
FRH-72	Specimen FR in an environment of 200 °C for 72 h
FRH-168	Specimen FR in an environment of 200 °C for 168 h
FRC-24	Specimen FR under the thermal cycle for 24 h
FRC-72	Specimen FR under the thermal cycle for 72 h
FRC-168	Specimen FR under the thermal cycle for 168 h

**Table 3:** EDX analysis results of points 1–6.

Point	Composition (at.%)	
	Al	Si
1	95.2	4.8
2	1.6	98.4
3	1.7	98.3
4	93.4	6.6
5	1.5	98.5
6	2.3	97.7

## Data Availability

The data presented in this study are available on request from the corresponding author.
